# Improved survival with second-line hepatic arterial infusion chemotherapy after atezolizumab-bevacizumab failure in hepatocellular carcinoma

**DOI:** 10.3389/fonc.2024.1495321

**Published:** 2024-12-12

**Authors:** Ji Yeon Lee, Jaejun Lee, Suho Kim, Jae-sung Yoo, Ji Hoon Kim, Keungmo Yang, Ji Won Han, Jeong Won Jang, Jong Yong Choi, Seung Kew Yoon, Ho Jong Chun, Jung Suk Oh, Pil Soo Sung

**Affiliations:** ^1^ Department of Internal Medicine, Seoul St. Mary’s Hospital, College of Medicine, The Catholic University of Korea, Seoul, Republic of Korea; ^2^ Division of Hepatology, Department of Internal Medicine, Seoul St. Mary’s Hospital, College of Medicine, The Catholic University of Korea, Seoul, Republic of Korea; ^3^ Department of Radiology, Seoul St. Mary’s Hospital, College of Medicine, The Catholic University of Korea, Seoul, Republic of Korea; ^4^ School of Medicine, Kyungpook National University, Daegu, Republic of Korea; ^5^ Division of Hepatology, Department of Internal Medicine, Uijeongbu St Mary’s Hospital, The Catholic University of Korea, Seoul, Republic of Korea

**Keywords:** carcinoma, hepatocellular, hepatic arterial infusion chemotherapy, atezolizumab, bevacizumab, immunogenic cell death, immunotherapy ` 2

## Abstract

**Background:**

There is no established second-line treatment for hepatocellular carcinoma (HCC) following atezolizumab-bevacizumab (ate-beva) failure. This study assessed the efficacy of hepatic arterial infusion chemotherapy (HAIC) as a salvage therapy by comparing survival outcomes and treatment responses between HAIC as a first-line treatment and as a second-line option after ate-beva failure.

**Materials and Methods:**

We retrospectively analyzed 100 patients with advanced HCC treated with HAIC between March 2022 and July 2024. Patients were categorized into two groups: those who received HAIC as initial therapy (first-line HAIC group) and those who received HAIC following ate-beva failure (post-ate-beva group). Survival outcomes were assessed with Kaplan-Meier curves and log-rank tests, and factors associated with survival were identified through Cox regression analysis.

**Results:**

The post-ate-beva group exhibited longer overall survival (OS) (median OS 12.4 months) compared to the first-line HAIC group (median OS 6.8 months) (p = 0.073). Progression-free survival (PFS) was significantly superior in the post-ate-beva group (median PFS 8.2 months) compared to the first-line HAIC group (median PFS 3.1 months) (p = 0.018). The objective response rate was also notably higher in the post-ate-beva group than in the first-line HAIC group (35.3% vs. 18.1%, p = 0.031). In multivariate analysis, HAIC following ate-beva failure, compared to first-line HAIC, was significantly associated with favorable outcomes for both OS (p = 0.014) and PFS (p = 0.006).

**Conclusion:**

The superior survival outcomes and treatment responses observed in the post-ate-beva group suggest that HAIC may be an effective second-line treatment option for advanced HCC following ate-beva therapy failure. However, due to the retrospective nature and small sample size of the study, further prospective studies with larger patient populations are needed to strengthen the evidence.

## 1 Introduction

Hepatocellular carcinoma (HCC) represents 80–90% of all primary liver cancers and is the sixth most commonly diagnosed cancer and the third leading cause of cancer-related deaths (1). Patients with advanced HCC (Barcelona Clinical Liver Cancer, BCLC Stage C) should be evaluated for systemic therapy (2). Since 2007, sorafenib, a multitarget tyrosine kinase inhibitor (MKI), has been the standard treatment for advanced HCC (3). Recent advances have included other MKIs, such as lenvatinib, regorafenib, and cabozantinib, and the vascular endothelial growth factor receptor 2 (VEGFR2) inhibitor, ramucirumab (4–7). Immune checkpoint inhibitors in combination with anti-VEGF agents, particularly a combination of atezolizumab and bevacizumab (ate-beva), have also emerged as first-line treatment options, demonstrating superiority over sorafenib in the IMbrave150 trial (8).

In this era of immunotherapy in HCC, several studies have explored the efficacy of various drugs as salvage or second-line therapies following ate-beva treatment (9–11). These include MKIs, such as lenvatinib, regorafenib, and cabozantinib, which have shown acceptable outcomes in a few real-world studies (12). Nivolumab plus ipilimumab was also studied for the effectiveness after other immune checkpoint inhibitor regimens (13). Despite these efforts, there is no consensus on the optimal second-line therapy after ate-beva, largely due to the relatively small sample sizes and limited high-quality evidence in these studies. Consequently, there remains an unmet need for further research to establish an effective second-line therapy after ate-beva failure.

Hepatic arterial infusion chemotherapy (HAIC) is a locoregional therapy for advanced HCC, mainly used in Asian countries, especially in Japan and South Korea (14, 15). HAIC delivers chemotherapeutic agents directly to liver lesions via the hepatic artery using a port system (16). Regimens, including cisplatin and 5-fluorouracil, also known as FP combination therapy, are strongly recommended for HAIC (17). The presence of intrahepatic tumors is a key prognostic factor for OS in patients with advanced HCC (18). In this context, HAIC has been shown to significantly extend patient survival by reducing intrahepatic tumor burden (19).

Furthermore, numerous studies have evaluated the efficacy of HAIC in patients with advanced HCC, compared to MKIs. In one study, no differences in OS and PFS were observed between patients treated with HAIC and lenvatinib for unresectable HCC (16). Subgroup analysis of patients with high tumor burden beyond the REFLECT eligibility criteria (e.g., tumor involvement > 50% of liver volume, main portal vein tumor thrombosis (PVTT), bile duct invasion) revealed that the HAIC group had significantly longer OS compared to the lenvatinib group (16). Another study, which focused on patients with advanced HCC and main PVTT, found that those treated with HAIC had a longer time-to-progression and DCR than those who were treated with sorafenib, with OS remaining comparable between two groups (20). A meta-analysis comparing HAIC and sorafenib in advanced HCC showed that HAIC was superior in terms of OS, PFS, ORR, and DCR (21). Overall, these findings suggest HAIC could be a valuable treatment approach, even in patients with advanced HCC.

While ate-beva has transformed the treatment landscape and improved prognosis for advanced HCC patients, no drug has yet been established as a definitive salvage therapy following ate-beva failure (22). HAIC, which has shown promising outcomes in HCC patients, is also being explored for its potential synergism with immunotherapy and VEGF inhibitors, raising its potential as a salvage therapy following ate-beva failure (23, 24). In the present study, we evaluated the efficacy of HAIC as a potential salvage therapy by comparing outcomes between first-line HAIC and HAIC administered as a second-line therapy after ate-beva failure.

## 2 Materials and methods

### 2.1 Patients

We retrospectively reviewed the electronic medical records of 100 patients in Seoul St. Mary’s Hospital with advanced HCC based on tumor staging with radiological and/or histological methods (25). Patients were categorized into two groups: those who initially received HAIC (first-line HAIC group) and those who received HAIC following ate-beva therapy (post-ate-beva group). Thirty-four patients transitioned from ate-beva therapy to HAIC due to tumor progression. Treatment failure with ate-beva was confirmed by identifying progressive disease (PD) using the modified Response Evaluation Criteria in Solid Tumors (mRECIST). This study was approved by the Institutional Review Board of the Catholic University of Korea (approval number: XC23RIDI0075) and was performed in accordance with the Declaration of Helsinki. Informed consent was waived due to the retrospective nature of the study.

### 2.2 Treatment

The ate-beva treatment protocol involves an infusion of 15 mg/kg of bevacizumab alongside 1200 mg atezolizumab every 3 weeks (8). In HAIC treatment, the insertion of an indwelling catheter in the hepatic artery and a subcutaneously implanted port system enables repeated intermittent administration of drugs (26). The HAIC protocol consists of a daily infusion of 5-fluorouracil (500 mg/m^2^) for three days, with cisplatin (60 mg/m^2^) being administered on the second day (26). Laboratory tests were performed daily during the 3 days of infusion time and were assessed for any severe adverse events arose from HAIC. If any kind of adverse events graded 3 or above according to the CTCAE were documented, clinicians decided whether to continue or interrupt the infusion schedule and take a day rest or terminate the session for this time. To prevent nausea, patients were administered with a 5-hydroxytryptamine 3 antagonist after the end of treatment. Each HAIC procedure was carried out by a team of interventional radiologists, each with more than 5 years of professional expertise. HAIC sessions were scheduled every 4–6 weeks unless the patient experienced disease progression or significant treatment-related side effects.

### 2.3 Response evaluation

Responses were categorized as complete response (CR), partial response (PR), stable disease (SD), or PD according to the mRECIST, and the ORR and DCR were assessed (27). CR was defined as the absence of arterial enhancement. PR was defined as a reduction of more than 30% in the sum of the diameters of viable tumors. PD was identified by more than a 20% increase in the diameter of viable lesions. SD refers to tumors that did not meet the criteria for PD or PR. The ORR was determined as the proportion of patients who maintained CR or PR for a minimum of 4 weeks after the initial radiological evaluation. The DCR was defined as the percentage of patients who achieved CR, PR, or SD. To assess treatment responses, all patients who received HAIC underwent follow-up imaging, including liver dynamic computed tomography scans or dynamic MRIs with liver-specific contrast agents after two or three cycles of therapy. Two independent radiologists independently performed response evaluation according to the mRECIST criteria.

### 2.4 Study endpoints

The primary endpoint of the study was OS, defined as the duration from the start of HAIC treatment to death from any cause. Patients who were lost to follow-up or remained alive at the end of the follow-up period were considered censored. The secondary endpoint of the study was PFS, defined as the duration from the start of HAIC treatment to disease progression or death from any cause.

### 2.5 Statistical analysis

For statistical analysis, R Statistical Software (v4.4.1; R Foundation Inc., Vienna, Austria; http://cran.r-project.org, accessed on August 1, 2024) was used. Categorical variables were compared using either Pearson’s chi-square test or Fisher’s exact test, depending on the expected frequency in each category. Independent t-tests were utilized for comparing continuous variables. Kaplan-Meier survival curves and log-rank tests were used to evaluate and compare the survival outcomes between the groups, respectively. Cox regression analyses were utilized to identify factors associated with survival outcomes. Variables with p-value < 0.1 in the univariate analysis were included in the multivariate analysis. Columns with missing values were excluded to ensure accurate statistical analysis. Statistical significance was defined as a p-value of < 0.05.

## 3 Results

### 3.1 Patient characteristics

The baseline characteristics of patients who received first-line HAIC (n = 66) and those who received HAIC after ate-beva therapy (n=34) are summarized in **Table 1**. A total of 100 patients were evaluated between March 2022 and June 2024. There were no significant differences between the two groups in terms of age (65.79 ± 11.19 vs. 62.12 ± 13.46 years, p = 0.151), sex distribution (p = 0.113), BCLC stage (p = 0.189), and Child−Pugh class (p = 0.285). Etiological factors, including hepatitis B virus (HBV), hepatitis C virus, alcohol use, and other causes, showed no significant differences between the two groups (p = 0.458). No significant differences were found between the groups in terms of ECOG performance status scores, serum AFP levels, and tumor size (p = 0.999, p = 0.815, and p = 0.624, respectively). The percentage of patients who had multiple tumors was significantly higher in the first-line HAIC group (p = 0.017). The presence of portal vein invasion and distant metastasis were also comparable between the two groups (p = 0.521 and p = 0.535, respectively).

**Table 1:** Patient demographics and characteristics.

	First-line HAIC (n = 66)	Post-Ate-beva HAIC (n = 34)	P-value
Age	65.79 ± 11.19	62.12 ± 13.46	0.151
Sex			0.113
Male	57 (86.4)	25 (73.5)	
Female	9 (13.6)	9 (26.5)	
AST (U/L)	98.26 ± 115.08	88.76 ± 77.39	0.62
ALT (U/L)	43.10 ± 39.62	49.26 ± 41.21	0.47
Total Bilirubin (mg/dL)	1.99 ± 3.61	1.23 ± 1.05	0.12
BCLC stage			0.189
0/A	6 (9.1)	1 (2.9)	
B	12 (28.2)	9 (26.5)	
C	48 (72.7)	22 (64.7)	
D	0 (0.0)	2 (5.9)	
Etiology			0.458
Hepatitis B virus	37 (56.0)	24 (70.6)	
Hepatitis C virus	6 (9.1)	3 (8.8)	
Alcohol	11 (16.7)	3 (8.8)	
Others	12 (28.2)	4 (11.8)	
Child–Pugh class			0.285
A	55 (83.3)	31 (91.2)	
B	8 (12.1)	3 (8.8)	
C	3 (4.5)	0	
ECOG performance status score			0.999
0	60 (90.9)	31 (91.2)	
1	4 (6.0)	2 (5.9)	
2	2 (3.0)	1 (2.9)	
Serum AFP level (ng/mL)	13505 ± 22884	12397 ± 21420	0.815
Tumor size (cm)	9.61 ± 4.82	9.08 ± 5.71	0.624
Number of tumors Single Multiple	14 (21.2) 52 (78.8)	15 (44.1) 19 (55.9)	0.017
Portal vein invasion			0.521
No	45 (68.2)	21 (61.8)	
Yes	21 (31.8)	13 (38.2)	
Distant metastasis			0.535
No	23 (34.8)	14 (41.2)	
Yes	43 (65.2)	20 (58.8)	
Prior treatments other than ate-beva treatment			< 0.001
TACE	0 (0.0)	13 (38.2%)	
Surgery	0 (0.0)	6 (17.6%)	
Systemic therapies	0 (0.0)	1 (2.9%)	
RT	0 (0.0)	4 (11.8%)	

Data are presented as n (%) and means ± standard deviations. Ate-beva, atezolizumab plus bevacizumab; HAIC, hepatic arterial infusion chemotherapy; BCLC stage, Barcelona Clinic Liver Cancer stage; ECOG performance status, Eastern Cooperative Oncology Group; AFP, alpha-fetoprotein. AST, aspartate aminotransferase; ALT, alanine aminotransferase; TACE, transarterical chemoembolization; RT, radiotherapy.

### 3.2 Survival outcomes

**Figure 1:**
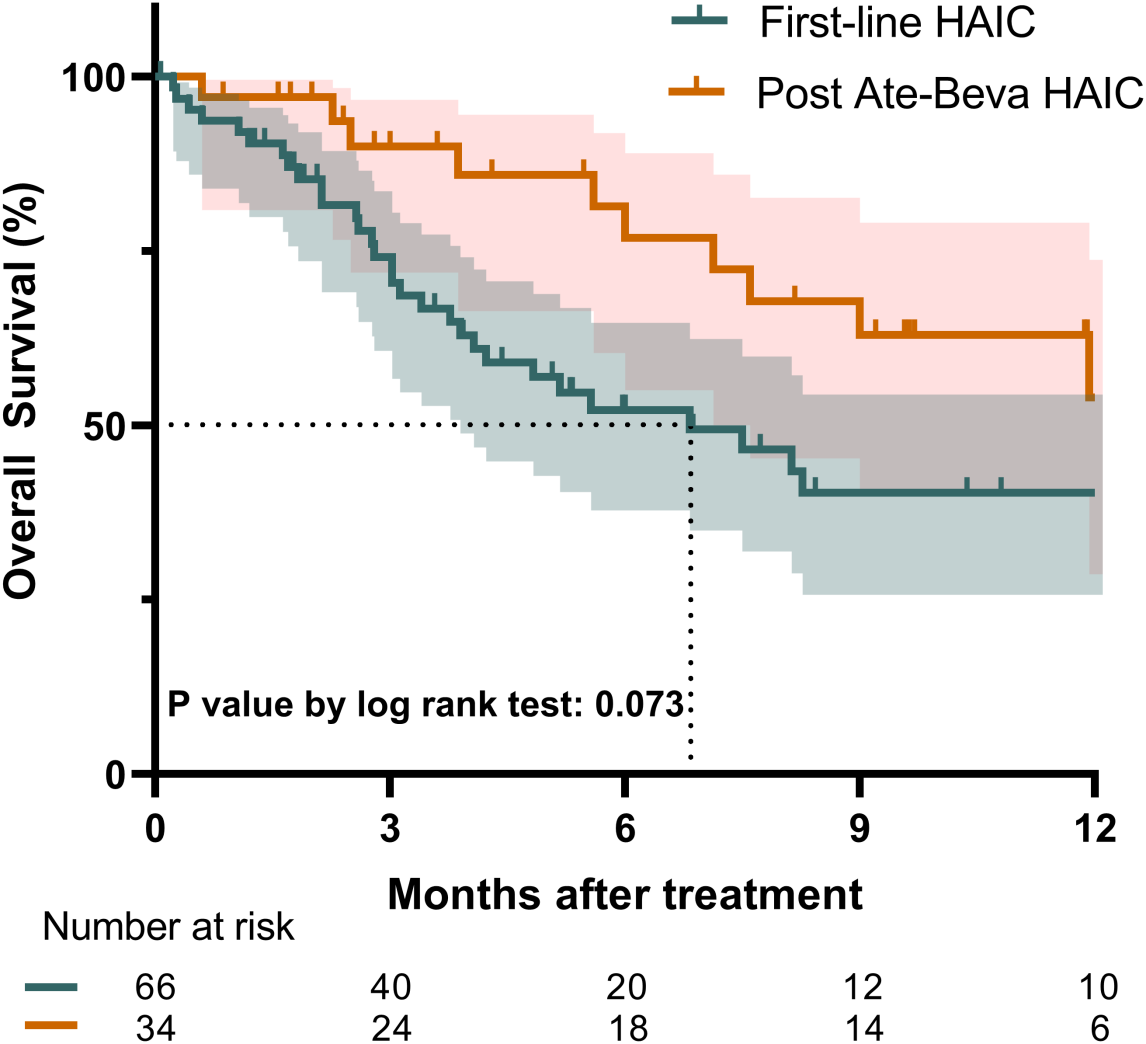
Overall survival comparison between patients treated with first-line HAIC and post-ate-beva HAIC using the Kaplan-Meier curve. The shaded area represents the 95% confidence interval. The dotted line indicates the median overall survival for each group, which exceeded 12 months for the post-ate-beva HAIC group and was 6.8 months for the first-line HAIC group. The number of patients at risk is displayed below the Kaplan-Meier curve. AB, Atezolizumab plus bevacizumab; HAIC, hepatic arterial infusion chemotherapy.

The median follow-up duration for the entire cohort was 4.3 months, with no significant differences observed between the two groups (p = 0.123). During the follow-up period, 47 deaths were recorded. The median OS for the entire study population was 8.3 months (95% confidence interval [CI]: 6.8–17.3). **Figure 1** illustrates the Kaplan-Meier survival curve for both groups. The post-ate-beva group demonstrated a longer median OS of 12.4 months (95% CI: 9.0, NA) compared to 6.8 months (95% CI: 4.1, NA) in the first-line HAIC group (p = 0.073). When comparing 12-month survival rates, the post-ate-beva group showed significantly higher survival rates of 54.0% (95% CI: 34.9–83.4) compared to 40.3% (95% CI: 28.0–58.1) in the first-line HAIC group (p = 0.043).

**Figure 2:**
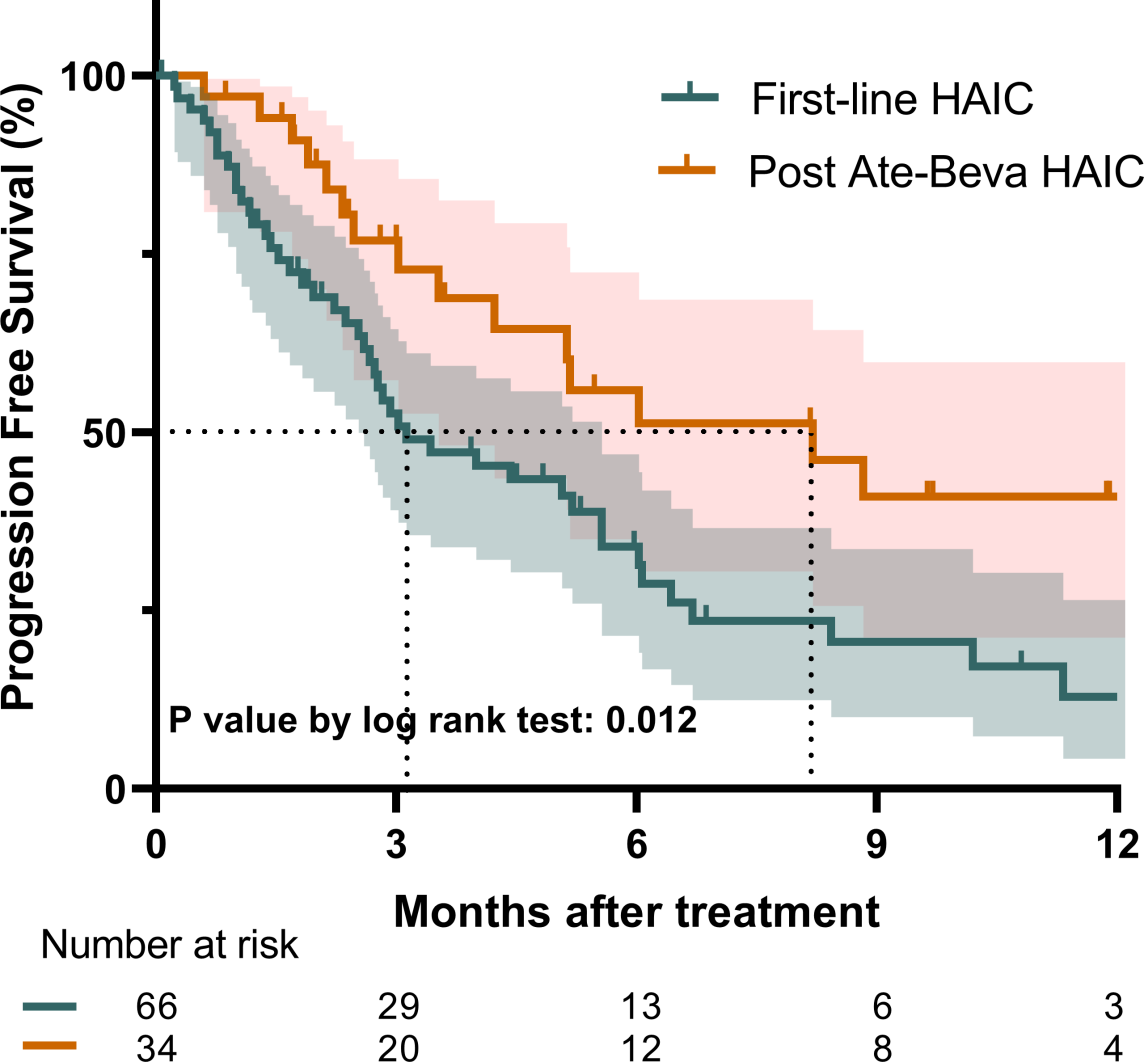
Progression-free survival comparison between patients treated with first-line HAIC and post-ate-beva HAIC using the Kaplan-Meier curve. The shaded area represents the 95% confidence interval. The dotted line indicates the median overall survival for each group, which was 8.2 months for the post-ate-beva HAIC group and was 3.1 months for the first-line HAIC group. The number of patients at risk is displayed below the Kaplan-Meier curve. AB, Atezolizumab plus bevacizumab; HAIC, hepatic arterial infusion chemotherapy.

Regarding PFS, the median PFS for the entire study population was 5.1 months (95% CI: 3.0–6.1). The post-ate-beva group demonstrated a significantly longer median PFS of 8.2 months (95% CI: 4.2, NA) compared to 3.1 months (95% CI: 2.7–5.6) in the first-line HAIC group (p = 0.018). The 12-month PFS rates also favored the post-ate-beva group, with a rate of 41.0% (95% CI: 25.0–67.1), compared to 12.9% (95% CI: 5.3–31.4) in the first-line group (p = 0.012) (**Figure 2**).

### 3.3 Treatment response

Tumor response to HAIC was assessed based on the best treatment responses (**Table 2**). Eleven patients in the first-line HAIC group had PR compared to 12 patients in the post-ate-beva HAIC group. The first-line HAIC group included 27 patients with SD, while the post-ate-beva HAIC group included 17 patients with SD. CR was observed in only one patient from the first-line HAIC group. The ORR was significantly higher in the post-ate-beva HAIC group at 35.3% (12 of 34 patients) compared to 18.1% (12 of 66 patients) in the first-line HAIC group (p = 0.031). Additionally, the DCR was significantly higher in the post-ate-beva HAIC group at 85.3% (29 of 34 patients) compared to 59.1% (39 of 66 patients) in the first-line HAIC group (p = 0.008).

**Table 2:** Treatment response evaluation.

Treatment responses	First-line HAIC (n = 66)	Post-ate-beva HAIC (n = 34)	P-value
			0.036
CR	1 (1.5%)	0 (0.0%)	
PR	11 (16.7%)	12 (35.3%)	
SD	27 (40.9%)	17 (50.0%)	
PD	19 (28.8%)	5 (14.7%)	
NA	8 (12.1%)	0 (0.0%)	
ORR	12 (18.1%)	12 (35.3%)	0.031
DCR	39 (59.1%)	29(85.3%)	0.008

Data are presented as n (%). Ate-beva, atezolizumab plus bevacizumab; HAIC, hepatic artery infusion chemotherapy; CR, complete response; PR, partial response; SD, stable disease; PD, progressive disease; NA, non-applicable; ORR, objective response rate; DCR, disease control rate.

### 3.4 Factors associated with survival outcomes

Factors associated with survival outcomes were assessed (**Table 3**). In terms of OS, univariate analysis revealed that an ECOG score of 0 (hazard ratio [HR] 0.233, 95% CI: 0.107–0.509), Child−Pugh Class A (HR 0.456, 95% CI: 0.242–0.859), and tumor size >10 cm (HR 1.982, 95% CI: 1.083–3.629) were significant factors. In multivariate analysis, the post-ate-beva HAIC compared to first-line HAIC (HR 0.404, 95% CI: 0.197–0.829, p = 0.014), along with an ECOG score of 0 (HR 0.265, 95% CI: 0.097–0.721, p = 0.009), were the only two factors associated with favorable OS outcomes in the study population.

**Table 3:** Factors associated with overall survival.

	Overall Survival			
	Univariate analysis		Multivariate analysis	
	HR (95% CI)	P-value	HR (95% CI)	P-value
Post ate-beva HAIC (vs. First-line HAIC)	0.560 (0.295−1.065)	0.077	0.404 (0.197−0.829)	0.014
Sex (Female)	1.353 (0.643−2.847)	0.426		
Etiology-Viral (vs. non-viral)	0.846 (0.454−1.575)	0.598		
ECOG 0	0.233 (0.107−0.509)	<0.001	0.265 (0.097−0.721)	0.009
Age >65 years	1.354 (0.749−2.449)	0.316		
Child-Pugh Class A	0.456 (0.242−0.859)	0.015	0.512 (0.251−1.045)	0.066
AFP >400ng/mL	1.698 (0.932−3.092)	0.084	1.767 (0.914−3.416)	0.090
Tumor size >10cm	1.982 (1.083−3.629)	0.027	1.208 (0.609−2.397)	0.588
Single mass (vs. multiple)	0.984 (0.553−1.751)	0.958		
PVTT	1.101 (0.597−2.033)	0.757		
Extrahepatic metastasis	1.667 (0.938−2.967)	0.081	1.049 (0.546−2.013)	0.886

Ate-beva, atezolizumab plus bevacizumab; HAIC, hepatic arterial infusion chemotherapy; BCLC stage, Barcelona Clinic Liver Cancer stage; ECOG performance status, Eastern Cooperative Oncology Group; AFP, alpha-fetoprotein.

Factors related to PFS were also analyzed (**Table 4**). In univariate analysis, post-ate-beva HAIC compared to first-line HAIC (HR 0.513, 95% CI: 0.292–0.901), ECOG score of 0 (HR 0.331, 95% CI: 0.155–0.708), Child−Pugh Class A (HR 0.549, 95% CI: 0.305–0.990), and the presence of extrahepatic metastasis (HR 2.079, 95% CI: 1.232–3.497) were significantly associated with PFS. In multivariate analysis, post-ate-beva HAIC (HR 0.441, 95% CI: 0.245–0.791, p = 0.006), ECOG score of 0 (HR 0.437, 95% CI: 0.196–0.974, p = 0.043), and the presence of extrahepatic metastasis (HR 1.753, 95% CI: 1.005–3.055, p = 0.048) remained significant factors influencing PFS.

**Table 4:** Factors associated with progression-free survival.

	Progression-Free Survival			
	Univariate analysis		Multivariate analysis	
	HR (95% CI)	P-value	HR (95% CI)	P-value
Post ate-beva HAIC (vs. First-line HAIC)	0.513 (0.292−0.901)	0.020	0.441 (0.245−0.791)	0.006
Sex (Female)	1.319 (0.709−2.454)	0.382		
Etiology-Viral (vs. non-viral)	1.084 (0.623−1.887)	0.775		
ECOG 0	0.331 (0.155−0.708)	0.004	0.437 (0.196−0.974)	0.043
Age >65 years	1.285 (0.771−2.140)	0.336		
Child−Pugh Class A	0.549 (0.305−0.990)	0.046	0.601 (0.321−1.125)	0.112
AFP >400ng/mL	1.247 (0.747−2.080)	0.398		
Tumor size >10cm	1.453 (0.870−2.428)	0.154		
Single mass (vs. multiple)	0.894 (0.543−1.472)	0.660		
PVTT	1.211 (0.709−2.066)	0.485		
Extrahepatic metastasis	2.079 (1.232−3.497)	0.006	1.753 (1.005−3.055)	0.048

Ate-beva, atezolizumab plus bevacizumab; HAIC, hepatic arterial infusion chemotherapy; BCLC stage, Barcelona Clinic Liver Cancer stage; ECOG performance status, Eastern Cooperative Oncology Group; AFP, alpha-fetoprotein.

## 4 Discussion

Recent studies have highlighted ate-beva therapy as a promising first-line option for patients with unresectable advanced HCC, demonstrating better outcomes compared to sorafenib [10]. However, the best second-line treatments for those whose disease progresses after initial ate-beva therapy are still not well established in HCC guidelines (28). Thus, there is an unmet need to explore second-line therapies for patients with HCC following ate-beva failure. In Asian countries, especially in South Korea and Japan, HAIC has been frequently used for unresectable advanced liver cancer because of its stronger antitumor effects compared to systemic chemotherapy and its reduced toxicity to other organs (29). However, there is still limited evidence on the effectiveness of HAIC following the failure of ate-beva therapy.

In the present study, we compared the treatment outcomes of HAIC administered after ate-beva treatment failure with first-line HAIC to evaluate the potential of HAIC as a salvage therapy in this setting. Our results showed that OS was longer in the post-ate-beva HAIC group than in the first-line HAIC group, though the difference did not reach statistical significance. This lack of significance may be attributed to the high proportion of censored data due to a relatively short follow-up period, which could dilute the observed differences between the groups. To address this, we also assessed the 12-month survival rates and found that patients who received HAIC following ate-beva treatment demonstrated significantly better 12-month OS and PFS rates compared to those who received HAIC as first-line therapy. Moreover, patients who received HAIC following ate-beva treatment exhibited higher ORR and DCR compared to those who received HAIC as their initial treatment. Multivariate analysis revealed that post-ate-beva HAIC, compared to the first-line HAIC, was a significant factor for favorable outcomes regarding OS and PFS.

Cancer cells evade immune surveillance by activating inhibitory mechanisms, often through the overexpression of specific checkpoint genes. Phagocytosis checkpoints like CD47, CD24, MHC-I, and PD-L1 play a critical role in cancer immunotherapy by acting as escape signals from immunogenic cells, thereby weakening the immune activity against tumors (30, 31). Given this background, the potential advantage of using HAIC as a second-line treatment following ate-beva failure might be explained by the immunogenic cell death (ICD) theory. ICD is associated with the release of various damage-associated molecular patterns (DAMPs) from dying cancer cells, including calreticulin, ATP, annexin A1, type 1 interferon, and high-mobility group box (32). These DAMPs interact with receptors on innate immune cells, such as pattern recognition receptors on dendritic cells, activate antigen-presenting cells, and initiate T-cell responses against cancer-specific antigens (33).

ICD also increases tumor-infiltrating lymphoid and myeloid cells, creating an immunoresponsive tumor microenvironment (TME) (34). Park et al. discovered that PD-L1 expressing tumor-associated macrophages (TAMs) are predominantly situated in the peritumoral area of HCC, and that blocking PD-L1 expression on macrophages could potentially restore the function of CD8+ and CD4+ T cells, hence improving the efficacy of immunotherapy (35). The combination of PD-1 inhibitors and anti-VEGF agents synergistically modulates the activity of effector T cells by normalizing the tumor vasculature within the TME. Anti-VEGF agent reduces VEGF-related immunosuppression in tumors and TMEs, and promotes T-cell infiltration, thereby enhancing the effectiveness of anti-PD-1 and anti-PD-L1 treatments, which strengthens antitumor immune response (36). This process helps transform “cold tumors” (which are less responsive to treatment) into “hot tumors” (which are more responsive). HAIC-based chemotherapy is known to facilitate the development of the TME, which is favorable for immunotherapy and boosting the antitumor effects of anti-PD-1 antibodies (37). Recent studies have also explored the use of peptides to specifically target oncogenic factors like PD-L1 and simultaneously address multiple factors, such as PD-L1 and VEGFR2, for more effective tumor suppression (38). In short, ICD induced by chemotherapy converts cancer cells into potent tumor vaccines, promoting the immune system’s ability to eliminate cancer cells, making it a valuable mechanism for cancer therapy (39). This synergy provides a strong rationale for using anti-PD-1 monoclonal antibodies and HAIC to treat advanced HCC (37).

In this context, several studies have explored the efficacy of cytotoxic chemotherapy in combination with an immune checkpoint inhibitor. Qin et al. demonstrated that combining camrelizumab (a PD-1 inhibitor) with oxaliplatin-based chemotherapy was tolerable in patients with advanced HCC and biliary tract cancer in terms of treatment response and safety (40). Another study conducted in China demonstrated that a regimen of camrelizumab, apatinib (a VEGFR-2 inhibitor), and HAIC was effective and safe for patients with BCLC stage C HCC (23). Zuo et al. also demonstrated that the combination of HAIC, camrelizumab, and apatinib resulted in superior OS and PFS in advanced HCC patients compared to treatment with camrelizumab and apatinib alone (41). Li et al. conducted a meta-analysis comparing the efficacy and safety of HAIC combined with immune checkpoint inhibitors and MKIs; the studies were divided into three groups of ICI plus other systemic therapies, HAIC therapy alone, and HAIC plus ICI or MKI therapy. The results showed that HAIC combined with ICI or MKI therapy demonstrated the longest median PFS of 9.37 months compared to other groups. Severe adverse effects were not significantly higher in the HAIC plus ICI or MKI group (42).

Despite the promising outcome of HAIC in our study, MKIs are the most favored second-line treatment following ate-beva treatments. In a multinational and multicenter retrospective study, Yoo et al. analyzed the clinical outcomes of MKIs after ate-beva failure, finding that second-line treatment with sorafenib and lenvatinib provided comparable efficacy and tolerable side effects (43). When comparing these two drugs in patients with advanced HCC after ate-beva failure, lenvatinib demonstrated superior PFS and comparable OS to sorafenib [11]. However, ORR was relatively low, with lenvatinib and sorafenib showing ORRs of 5.6% and 8.3%, respectively, highlighting their limitation as second-line treatment after ate-beva failure. These studies also include a relatively small number of patients, and differences in baseline characteristics were not sufficiently controlled, limiting the accuracy of the comparisons (43–45). The absence of a well-structured, randomized controlled trial reduces the level of evidence supporting the use of MKIs after ate-beva failure. Additionally, studies assessing the efficacy of MKIs combined with ICI therapy revealed more frequent and severe toxicities, raising concerns about the safety of these combination therapies (46). In this context, further studies are required to explore suitable second-line therapies for specific patient populations to better guide clinicians in making personalized treatment decisions, with HAIC being considered as a potential option.

Our study had several limitations. First, it had a retrospective design, which resulted in unequal distributions in both groups and may have introduced confounding factors. Future studies with randomized control trials or those utilizing propensity score matching would enhance the reliability of the study results. Second, the study predominantly involved an East Asian population with a high prevalence of HBV infection, which may limit the applicability of the results to other ethnicities and regions. Third, although OS was longer in the post-ate-beva group, the difference between the two groups was not statistically significant. A study with a longer follow-up period would be beneficial to further clarify the OS differences between these groups. Finally, the sample size was relatively small. To further strengthen this evidence, future studies should have prospective designs and include larger and more diverse populations, with baseline characteristics adjustments.

In conclusion, our results demonstrated that OS, PFS, ORR, and DCR were superior in patients with advanced HCC who received HAIC following ate-beva failure compared to those who received HAIC as an initial treatment. These findings suggest that HAIC may be a promising second-line treatment option for advanced HCC after ate-beva failure. Further studies comparing the treatment outcome of HAIC to other MKIs as a second-line treatment are required to determine the optimal therapy after the failure of ate-beva treatment.

## Data Availability

The raw data supporting the conclusions of this article will be made available by the authors, without undue reservation.
